# Cardiac dose reduction with deep inspiration breath hold for left-sided breast cancer radiotherapy patients with and without regional nodal irradiation

**DOI:** 10.1186/s13014-015-0511-8

**Published:** 2015-09-22

**Authors:** Rosanna Yeung, Leigh Conroy, Karen Long, Daphne Walrath, Haocheng Li, Wendy Smith, Alana Hudson, Tien Phan

**Affiliations:** Division of Radiation Oncology, Department of Oncology, University of Calgary,, 2500 University Dr NW, Calgary, Alberta T2N 1 N4 Canada; Department of Medical Physics, Tom Baker Cancer Centre, 1331 29 Street Northwest, Calgary, AB T2N 4 N2 Canada; Department of Physics & Astronomy, University of Calgary, 2500 University Dr NW, Calgary, Alberta T2N 1 N4 Canada; Department of Radiation Therapy, Tom Baker Cancer Centre, 1331 29 Street Northwest, Calgary, AB T2N 4 N2 Canada; Departments of Oncology and Community Health Sciences, University of Calgary, 2500 University Dr NW, Calgary, Alberta T2N 1 N4 Canada; Division of Medical Physics, Department of Oncology, University of Calgary, 2500 University Dr NW, Calgary, Alberta T2N 1 N4 Canada

**Keywords:** Breast cancer, Deep inspiration breath hold, Dosimetry, Regional nodal irradiation, Patient selection, Cardiac dose

## Abstract

**Background:**

Deep inspiration breath hold (DIBH) reduces heart and left anterior descending artery (LAD) dose during left-sided breast radiation therapy (RT); however there is limited information about which patients derive the most benefit from DIBH. The primary objective of this study was to determine which patients benefit the most from DIBH by comparing percent reduction in mean cardiac dose conferred by DIBH for patients treated with whole breast RT ± boost (WBRT) versus those receiving breast/chest wall plus regional nodal irradiation, including internal mammary chain (IMC) nodes (B/CWRT + RNI) using a modified wide tangent technique. A secondary objective was to determine if DIBH was required to meet a proposed heart dose constraint of D_mean_ < 4 Gy in these two cohorts.

**Methods:**

Twenty consecutive patients underwent CT simulation both free breathing (FB) and DIBH. Patients were grouped into two cohorts: WBRT (*n* = 11) and B/CWRT + RNI (*n* = 9). 3D-conformal plans were developed and FB was compared to DIBH for each cohort using Wilcoxon signed-rank tests for continuous variables and McNemar’s test for discrete variables. The percent relative reduction conferred by DIBH in mean heart and LAD dose, as well as lung V_20_ were compared between the two cohorts using Wilcox rank-sum testing. The significance level was set at 0.05 with Bonferroni correction for multiple testing.

**Results:**

All patients had comparable target coverage on DIBH and FB. DIBH statistically significantly reduced mean heart and LAD dose for both cohorts. Percent reduction in mean heart and LAD dose with DIBH was significantly larger in the B/CWRT + RNI cohort compared to WBRT group (relative reduction in mean heart and LAD dose: 55.9 % and 72.1 % versus 29.2 % and 43.5 %, *p* < 0.02). All patients in the WBRT group and five patients (56 %) in the B/CWBRT + RNI group met heart D_mean_ <4 Gy with FB. All patients met this constraint with DIBH.

**Conclusions:**

All patients receiving WBRT met D_mean_ Heart < 4 Gy on FB, while only slightly over half of patients receiving B/CWRT + RNI were able to meet this constraint in FB. DIBH allowed a greater reduction in mean heart and LAD dose in patients receiving B/CWRT + RNI, including IMC nodes than patients receiving WBRT. These findings suggest greatest benefit from DIBH treatment for patients receiving regional nodal irradiation.

## Background

Adjuvant radiation therapy (RT) for breast cancer patients reduces the risk of local recurrence and improves overall survival [1, 2]. RT to the left breast and chest wall results in non-negligible dose to the heart and coronary arteries. Several large studies have demonstrated increased cardiac mortality associated with RT for left-sided breast cancer [3–5]. The use of cardiotoxic chemotherapy may further increase this risk [6].

Techniques to minimize irradiation of cardiac structures without compromise to target coverage have been developed including the use of deep inspiration breath hold (DIBH). While there is not yet any clinical evidence showing reduced cardiac toxicity or morbidity when using DIBH, numerous planning studies have demonstrated decreased dose to cardiac structures when compared to FB [7–15]. However, threshold doses to the heart and coronary arteries have not been determined [13]. A recent international breast cancer randomized trial (NSABP B-51/RTOG1304), has proposed that the mean heart dose (D_mean_ Heart) should be <4 Gy during left-sided breast/chest wall irradiation [16]. Dose limits have not been established for the left-anterior descending coronary artery (LAD), which may be the crucial target to mitigate ischemic heart disease risk from RT [17, 18].

DIBH is not uniformly implemented. A survey conducted by the authors in May 2014 identified that only four of fourteen cancer centers in Western Canada were using DIBH during RT for left-sided breast cancer, in part because of concerns about technical complexity and infrastructure constraints. Our institution implemented a visually monitored DIBH technique in June 2013 to avoid challenges with limited access to respiratory gating devices on older linear accelerators and to reduce simulation and treatment delivery time for DIBH.

The primary objective of this study was to identify which patients would benefit most from DIBH by determining whether a difference in percent mean cardiac dose reduction exists in patients receiving whole breast radiotherapy alone compared to those also receiving regional nodal irradiation (RNI), including internal mammary chain (IMC) nodal RT using a modified wide tangent technique. Our secondary objective was to determine if DIBH was required to meet the proposed mean heart dose constraint of <4 Gy.

## Methods

### Patient population

Twenty consecutive patients with left-sided breast cancer treated at our center starting in June 2013 were included if they were able to maintain DIBH ≥ 20 seconds. All patients received CT simulation with standard FB and DIBH and were treated on a wing-board. The DIBH technique involved training patients immediately prior to CT simulation. The distances from table-top to the mid-axillary line in FB and DIBH were recorded and displacements were calculated. Once reproducibility in DIBH was established based on these measurements, patients were translated through the CT with bellows (Philips, The Netherlands) to ensure stability of breath hold.

Clinical target volumes (CTV) included the breast or chest wall (CTV_b_) for all patients. If the regional nodes were to be treated, additional CTVs for the internal mammary chain nodes inclusive of the first to third interspaces (CTV_IMC_) and axillary plus supraclavicular nodes were delineated. CTV_b_ was based on RTOG guidelines [19]. IMC nodes were delineated by a 1 cm diameter circle around the internal mammary vessels from the first to third intercostal space, and cropped from lung and bone [20]. If boost to the seroma was deemed necessary, PTV_boost_ was delineated by expanding the seroma contour by 1 cm in all directions. Organs at risk (OAR) were defined as the heart, LAD, and left lung. The heart and LAD were contoured based on previously published guidelines [21].

### Treatment planning

For each patient, 3D-CRT plans on the FB and DIBH scans were developed using forward planning, field-based techniques. Tangential medio-lateral opposed fields with dynamic wedges were used to cover the CTV_b_ ± CTV_IMC_. IMC nodes were covered using a modified wide tangent technique [20] where tangent fields were widened to include the CTV_IMC_ and narrowed inferiorly to reduce lung and cardiac doses. Supraclavicular and axillary nodes were covered by a single anterior field, or opposed anterior-posterior fields as determined by the treating radiation oncologist. The aim of the plans was to cover ≥ 95 % of the CTV_b_ ± supraclavicular/axillary nodes with ≥95 % of the prescription dose. If CTV_IMC_ was included, target coverage of ≥80 % of the prescription dose to 100 % of the volume was used.

### Dosimetric evaluation

CTV_b_ volumes, D_95_ coverage of the CTV_b_, and IMC coverage (if treated) on FB and DIBH scans for each patient were recorded. Heart V_25_, left lung V_20_, and mean doses (D_mean_) to the heart and LAD on FB and DIBH scans were determined. Percent dose reduction conferred by DIBH was calculated for mean heart and mean LAD doses as well as left lung V_20_ for each patient.

### Statistical analysis

Patients were classified into two treatment cohorts: patients who received whole breast radiotherapy alone ± boost (WBRT), and patients who received breast or chest wall radiotherapy plus regional nodal irradiation, including the IMC and supraclavicular nodes (B/CWRT + RNI). Results were analyzed using R Software version 3.1.2 [22]. To evaluate FB vs. DIBH within each cohort, Wilcoxon signed-rank tests were used for continuous variables and McNemar’s test was used to test differences for discrete variables. The percent relative reduction when using DIBH as opposed to FB was compared between the two cohorts for mean heart dose, mean LAD dose, and lung V_20_ using Wilcoxon rank-sum tests. The Bonferroni correction method was used to correct for multiple comparisons. The initial significance level of 0.05 was divided by the number of tests to obtain a modified significance level.

## Results

### Demographics

Eleven patients received WBRT and nine patients received B/CWRT + RNI. Table 1 shows the subjects’ baseline demographics and radiotherapy treatment characteristics. Stage distribution dictated the treatment technique and therefore, was different between cohorts. Dose and fractionation prescribed in this study represents standard treatment prescriptions at our institution. The most common WBRT dose fraction was 42.5 Gy in 16 fractions. For patients receiving additional RNI, dose fractionation ranged from 40–50 Gy in 16–25 fractions to the breast or chest wall and 37.5 Gy-45 Gy in 16–25 fractions to regional nodes.

**Table 1:** Baseline demographic and radiotherapy treatment parameters for left-sided breast cancer patients by treatment cohort

Characteristics	WBRT (*n* = 11) (%)	B/CWRT + RNI (*n* = 9) (%)
Median age (years), range	47 (39–54)	51 (34–69)
AJCC Stage		
DCIS	3 (27)	0 (0)
I	5 (45)	0 (0)
II	3 (27)	5 (55)
III	0 (0)	4 (44)
ER/PR positive (for invasive disease)	7/8 (88)	7/9 (78)
HER 2+ (for invasive disease)	2/8 (25)	2/9 (22)
Surgery		
Breast conserving	11 (100)	5 (56)
Mastectomy	0 (0)	4 (44)
RT boost to seroma	4 (36)	2 (22)
RT to internal mammary nodes	0 (0)	9 (100)
Breast/Chest wall RT dose		
(Gy/# fraction)		
40/16	0 (0)	2 (22)
42.5/16	10 (91)	4 (44)
45/25	0 (0)	1 (11)
50/25	1 (9)	2 (22)
Supraclavicular nodal RT dose		
(Gy/# fraction)		
37.5/16	N/A	6 (67)
45/25	N/A	3 (33)

### Dosimetric outcomes

**Fig. 1:**
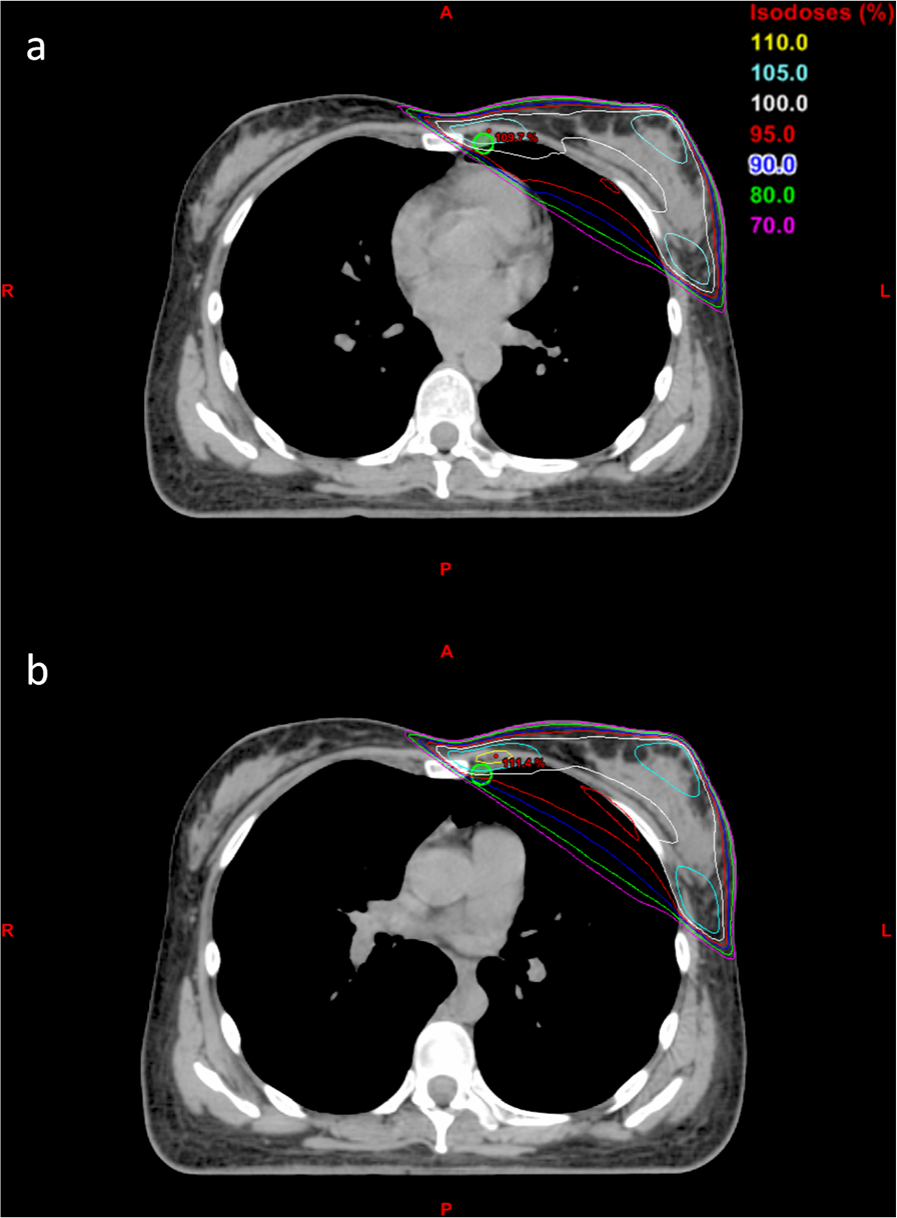
CT simulation axial images of one patient at (**a**) FB and (**b**) DIBH with treatment plan isodose lines. The IMC nodes contour is shown in green

Figure 1 shows a CT planning axial image through the same portion of the left breast in FB (Fig. 1a) and DIBH (Fig. 1b) in a patient with the IMC nodes included in the 80 % breast prescription isodose line. Comparison of average dose parameters to target volumes and OARs for FB and DIBH are summarized in Table 2. The average D_95_ coverage of CTV_b_ for the B/CWRT + RNI group in FB was slightly lower (92.2 ± 8.1 %) than the planning dose constraint of D_95_ >95 %_._ However, after correcting for multiple comparisons D_95_ of the CTV_b_ was not significantly different between FB and DIBH for both cohorts. Patients in the B/CWRT + RNI group achieved comparable IMC coverage with FB and DIBH planning (average V_80_ for CTV_IMC_ 99.7 % and 98.9 % for FB and DIBH respectively, *p* = 1.000). Mean CTV_b_ volume was comparable for FB vs. DIBH in both cohorts.

**Table 2:** Comparison of average dose parameters for targets and OARs for left-sided breast cancers by treatment cohort

Parameter	WBRT	WBRT FB vs. DIBH *p*-value*	B/CWRT + RNI	B/CWRT FB vs. DIBH *p*-value***	All Patients	FB vs. DIBH *p*-value*
CTV_b_ Volume (cc)						
FB	515.5 ± 356.9	0.577	489.2 ± 200.8	0.496	503.7 ± 290.2	0.349
DIBH	511.8 ± 354.6		491.3 ± 218.6		502.6 ± 294.0	
D_95_ for CTV_b_ (%)						
FB	97.3 ± 1.4	0.286	92.2 ± 8.1	0.030	95.0 ± 5.9	0.013
DIBH	97.7 ± 1.3		96.2 ± 3.0		97.0 ± 2.3	
V_80_ IMC (%)						
FB	N/A	N/A	99.7 ± 0.9	1.000	N/A	N/A
DIBH	N/A		98.9 ± 3.3		N/A	
D_mean_ Heart (cGy)						
FB	166.1 ± 71.0	0.001**	375.7 ± 186.0	0.004**	260.5 ± 169.3	<0.001**
DIBH	110.8 ± 32.1		145.0 ± 37.7		126.2 ± 38.0	
V_25_ Heart (%)						
FB	0.8 ± 1.4	0.059	4.0 ± 3.4	0.014	2.2 ± 2.9	0.002**
DIBH	0.1 ± 0.4		0.0 ± 0.1		0.1 ± 0.3	
D_mean_ LAD (cGy)						
FB	1004.6 ± 892.4	0.002**	1802.8 ± 917.8	0.004**	1363.8 ± 969.4	<0.001**
DIBH	420.0 ± 277.4		396.2 ± 81.5		409.3 ± 208.4	
V_20_ Left Lung (%)						
FB	10.5 ± 4.6	0.067	18.3 ± 5.1	0.250	14.0 ± 6.2	0.029
DIBH	8.5 ± 3.6		16.3 ± 2.8		12.0 ± 5.1	
Patients meeting						
D_mean_ < 4Gy (fraction, %)						
FB	11/11 (100)	N/A	5/9 (56)	0.134	16/20 (80)	0.134
DIBH	11/11 (100)		9/9 (100)		20/20 (100)	

Wilcoxon signed-rank test is used for comparison of all continuous variables, and McNemar's test is applied for discrete variables

Significant *p*-values after adjusting for multiple testing are indicated by a double asterisk (**)

Abbreviations: WBRT = whole breast RT ± boost cohort, B/CWRT + RNI = breast or chest wall + regional nodal RT cohort, FB = free-breathing, DIBH = deep inspiration breath hold, CTV_b_ = CTV for breast or chest wall, IMC = internal mammary chain nodes, D_95_ = Dose to 95 % target volume,V_80_ = % volume of organ receiving 80 % of the prescription dose, V_25_ = % volume of organ receiving 25 Gy, D_mean_ = Mean dose, LAD = left anterior descending coronary artery, V_20_ = % volume of organ receiving 20 Gy, N/A = not applicable. Data shown are mean values with one standard deviation. *In this group, to achieve significance p-values must be < 0.0083 to account for multiple testing. ***In this group, to achieve significance *p*-values must be < 0.0071 to account for multiple testing. **Indicates significance when compared to the Bonferroni adjusted criterion

The DIBH technique significantly reduced D_mean_ Heart compared to FB for both the WBRT (*p *= 0.001) and B/CWRT + RNI (*p* = 0.004) groups. DIBH also significantly reduced D_mean_ LAD in both treatment cohorts (*p* < 0.005 for both) when compared to FB. Heart V_25_ was not significantly reduced with DIBH in either of the two cohorts alone; while the combined group of all patients showed a significant difference (*p* = 0.002). There was no significant difference between FB and DIBH in left lung V_20_ for either cohort alone or for all patients combined.

All patients in the WBRT group were able to meet a D_mean_ Heart of <4 Gy on FB planning, but only five of nine patients (56 %) in the B/CWRT + RNI group were able to meet this constraint with FB. With DIBH, all patients in both cohorts were able to meet the D_mean_ Heart <4 Gy constraint.

Average relative percent reduction per patient in OAR dose parameters with DIBH are presented in Table 3. While Table 2 demonstrates that both groups of patients benefit from DIBH, the data presented in Table 3 shows that patients receiving regional nodal irradiation achieved a statistically significantly greater percent reduction in D_mean_ Heart and LAD from DIBH then those receiving WBRT alone. The reduction in left lung V_20_ with DIBH was modest (<10 %) and not significantly different between WBRT and B/CWRT + RNI groups.

**Table 3:** Average patient percent relative reduction in dose parameters with DIBH compared to FB for left-sided breast cancer patients by treatment cohort. Significant *p*-values after adjusting for multiple testing are indicated by a double asterisk (**)

Parameter	WBRT	B/CWRT + RNI	*p*-value*
D_mean_ Heart	29.2 %	55.9 %	0.003**
D_mean_ LAD	43.5 %	72.1 %	0.014**
V_20_ Left Lung	8.9 %	6.6 %	0.305

Wilcoxon sum-rank test used in for comparison of all variables*In this group, to achieve significance p-values must be < 0.0167 to account for multiple testing. **Indicates significance when compared to the Bonferroni adjusted criterion

## Discussion

There is a wealth of evidence from retrospective and planning studies demonstrating reduction in dose to the heart and coronary arteries with DIBH treatment of left-sided breast cancers. However, most published large retrospective studies have combined all patients, irrespective of regional nodal irradiation in their analysis [8]. Similarly, few published planning studies have compared dosimetric outcomes between patients receiving WBRT alone (without IMC treatment) and those requiring additional regional nodal irradiation including treatment of the IMC nodes [7–14]. A planning study from Toronto demonstrated that DIBH reduced heart dose for some patients treated with wide breast tangents but included only five patients [15]. The current study is one of the larger studies evaluating differences in OAR dose reductions with DIBH comparing WBRT alone to patients receiving breast/chest wall plus regional nodal irradiation employing a modified wide tangents technique to include the IMC nodes.

In this study, the average CTV_b_ volumes and D_95_ coverage with DIBH were comparable to FB within each cohort, confirming minimal bias in regards to volume delineation and planning. The study results are similar to published retrospective and planning studies demonstrating that DIBH lowered D_mean_ Heart and LAD doses [7–15]. Planning studies have shown variable results regarding the impact of DIBH on ipsilateral lung dose-volume relationships. Some authors have reported that DIBH significantly reduced lung dose while others showed no difference [9–13]. In the current study, DIBH did not significantly reduce left lung V_20._ It is possible that the small sample sizes used in this study, however, did not achieve the statistical power to show this effect. Further studies with larger sample sizes are required to determine if there is a statistical difference in lung V_20_ between FB and DIBH.

DIBH significantly reduced both D_mean_ Heart and LAD doses in both treatment cohorts. A literature review of clinical and dose volume predictors for radiation-induced heart disease found no consensus regarding a safe threshold dose for the heart or LAD [22]. However, the ongoing NSABP-B-51/RTOG1104 study protocol has recommended a D_mean_ Heart constraint for left sided breast/chest wall irradiation of <4 Gy [16]. Although there is evidence to suggest no safe threshold dose to the heart, the proposed dose of <4 Gy served as a reference to enable comparison in the current study [4,23]. All patients in the WBRT group were able to meet a D_mean_ Heart <4Gy with FB while only slightly over half of the patients receiving regional nodal irradiation were able to meet this constraint with FB. All patients were able to meet this constraint with DIBH, suggesting that DIBH provides a greater benefit for patients receiving regional nodal irradiation when using a modified wide tangent technique. Similarly, the relative percent reduction in D_mean_ Heart and LAD dose with DIBH was significantly greater in the B/CWRT + RNI group compared to the WBRT group. To improve treatment planning efficiency, these observations have led to an institutional policy to use DIBH in all patients with left-sided breast cancer when the RT intent is to include the regional lymph nodes, but to use it selectively if the RT intent is whole breast RT alone.

In this study a modified wide tangent technique was used for IMC nodal irradiation. Results from some treatment planning studies have suggested that partially-wide tangent techniques may provide superior dosimetric coverage of the IMC nodes compared to photon-electron techniques [24, 25]. The disadvantage of using modified wide tangents for IMC node irradiation is the potentially increased dose to the heart and lung; however we have shown here that DIBH significantly reduces this effect. The relative effectiveness of DIBH for patients receiving alternate forms of regional nodal irradiation, such as the photon-electron technique, is not examined in this study.

A limitation of the current study is the relatively small sample size. However, the patients were consecutive eligible cases, so the findings may more readily translate to other populations. Furthermore, all patients treated to the regional lymph nodes also had the IMC nodes included. Whether DIBH would be needed as frequently if the IMC nodes were excluded could not be addressed by the current data. However, the report of the EORTC study showing a survival advantage for the use of IMC plus medial supraclavicular RT may increase the frequency of IMC treatment [26]. Both D_mean_ Heart and D_mean_ LAD were significantly reduced in both groups. One may argue that the LAD is a more important target to avoid in the pathogenesis of long term cardiac complications [14, 27]. Further studies are required to determine threshold doses to cardiac structures in breast radiotherapy.

## Conclusions

DIBH provided greater percent reductions in mean heart and LAD doses for patients receiving regional nodal irradiation that included the IMC nodes using a modified wide tangent technique than for whole breast RT alone. All patients receiving WBRT alone met the mean heart dose constraint of <4 Gy on free breathing planning, while only slightly over half of patients receiving regional nodal irradiation were able to meet this constraint in free breathing. DIBH is justified for all patients receiving RT for left-sided breast cancer, but as a minimum, should be used regularly for all left-sided breast cancer patients receiving breast/chest wall RT plus nodal RT.

## References

[1] Clarke M, Collins R, Darby S, Davies C, Elphinstone P, Evans V (2005). Effects of radiotherapy and of differences in the extent of surgery for early breast cancer on local recurrence and 15-year survival: an overview of the randomized trials. Lancet.

[2] McGale P, Taylor C, Correa C, Cutter D, Duana F, EBCTCG (Early Breast Cancer Trialists' Collaborative Group) (2014). Effect of radiotherapy after mastectomy and axillary surgery on 10-year recurrence and 20-year breast cancer mortality: meta-analysis of individual patient data for 8135 women in 22 randomised trials. Lancet.

[3] Paszat LF, Mackillop WJ, Groome PA, Schulze K, Holowaty E (1999). Mortality from myocardial infarction following post-lumpectomy radiotherapy for breast cancer: a population-based study in Ontario, Canada. Int J Radiat Oncol Biol Phys.

[4] Darby SC, Ewertz M, McGale P, Bennet AM, Blom-Goldman U, Bronnum D (2013). Risk of ischemic heart disease in women after radiotherapy for breast cancer. N Eng J Med.

[5] Giordano SH, Kuo YF, Freeman JL, Buchholz TA, Hortobaqyi GN, Goodwin JS (2005). Risk of cardiac death after adjuvant radiotherapy for breast cancer. J Natl Cancer Inst.

[6] Shapiro CL, Hardernbergh PH, Gelman R, Blanks D, Hauptman P, Recht A (1998). Cardiac effects of adjuvant doxorubicin and radiation therapy in breast cancer patients. J Clin Oncol.

[7] Hjelstuen MHB, Mjaaland I, Vikström J, Dybvik KI (2012). Radiation during deep inspiration allows loco-regional treatment of left breast and axillary-, supraclavicular and internal mammary lymph nodes without compromising target coverage or dose restrictions to organs at risk. Acta Oncol.

[8] Nissen HD, Appelt AL (2013). Improved heart, lung, and target dose with deep inspiration breath hold in a large clinical series of breast cancer patients. Radiother Oncol.

[9] Korreman SS, Pedersen AN, Nøttrup TJ, Specht L, Nyström H (2005). Breathing adapted radiotherapy for breast cancer: comparison of free breathing gating with the breath-hold technique. Radiother Oncol.

[10] Pedersen AN, Korreman S, Nyström H, Specht L (2004). Breathing adapted radiotherapy of breast cancer: reduction of cardiac and pulmonary doses using voluntary inspiration breath hold. Radiother Oncol.

[11] Remouchamps VM, Vicini FA, Sharpe MB, Kestin LL, Martinez AA, Wong JW (2003). Significant reductions in heart and lung doses using deep inspiration breath hold with active breathing control and intensity modulated radiation therapy for patients treated with locoregional breast irradiation. Int J Radiat Oncol Biol Phys.

[12] Hayden A, Rains M, Tiver K (2012). Deep inspiration breath hold technique reduces heart dose from radiotherapy for left-sided breast cancer with deep breath-holding. J Med Imaging Radiat Oncol.

[13] Nemoto K, Ogushi M, Nakajima M, Kozuka T, Nose T, Yamashita T (2009). Cardiac-sparing radiotherapy for left breast cancer with deep breath-holding. Jpn J Radiol.

[14] Lu HM, Cash E, Chen MH, Chin L, Manning WJ, Harris J (2000). Reduction of cardiac volume in left-breast treatment field by respiratory maneuvers: a CT study. Int J Radiat Oncol Biol Phys.

[15] Sixel K, Aznar M, Ung Y (2001). Deep inspiration breath hold to reduce irradiated heart volume in breast cancer patients. Int J Radiat Oncol Biol Phys.

[16] Breast NSA, Project B. NSABP Protocol B-51: A randomized phase III clinical trial evaluating post-mastectomy chestwall and regional nodal xrt and post-lumpectomy regional nodal xrt in patients with positive axillary nodes before neoadjuvant chemotherapy who convert to pathologically negative axillary nodes after neoadjuvant chemotherapy. Pittsburgh: NSABP; 2013 [Available at: mtcancer.org/Protocols/B51_Protocol.pdf; cited April 25, 2014].

[17] Sadaro A, Petruzzelli MF, D’Errico MP, Grimauldi L, Pili G, Portaluri M (2012). Radiation-induced cardiac damage in early left breast cancer patients: risk factors, biological mechanisms, radiobiology, and dosimetric constraints. Radiother Oncol.

[18] Nilsson G, Holmberg L, Carmo H, Duvernoy O, Sjögren I, Lagerqvist B (2012). Distribution of coronary artery stenosis after radiation for breast cancer. J Clin Oncol.

[19] White J, Tai A, Arthur D, Buchholz T, MacDonald S, Marks L, et al. Radiotherapy Oncology Group breast cancer atlas for radiation therapy planning: consensus definitions. [Available at: http://www.rtog.org/LinkClick.aspx?fileticket=vzJFhPaBipE%3d&tabid=23; cited June 15, 2013].

[20] Marks LB, Hebert ME, Bentel G, Spencer DP, Sherouse GW, Prosnitz LR (1994). To treat or not to treat the internal mammary nodes: a possible compromise. Int J RadiatOncolBiolPhys.

[21] Feng M, Moran JM, Koelling T, Chughtai A, Chan JL, Freedman L (2011). Development and validation of a heart atlas to study cardiac exposure to radiation following treatment for breast cancer. Int J Radiat Oncol Biol Phys.

[22] R Core Team (2015). R: A language and environment for statistical computing. R Foundation for Statistical Computing. Vienna, Austria. http://www.R-project.org. Accessed 01 Sept 2015.

[23] Gagliardi G, Constine LS, Moiseenko V, Correa C, Pierce LJ, Allen AM (2010). Radiation Dose-Volume Effects in the Heart. Int J Radiat Oncol Biol Phys.

[24] Arthur DW, Arnfield MR, Warwicke LA, Morris MM, Zwicker RD (2000). Internal mammary node coverage: an investigation of presently accepted techniques. Int J Radiat Oncol Biol Phys.

[25] Severin D, Connors S, Thompson H, Rathee S, Stavrev P, Hanson J (2003). Breast radiotherapy with inclusion of internal mammary nodes: a comparison of techniques with three-dimensional planning. Int J Radiat Oncol Biol Phys.

[26] Poortmans P, Struikmans H, Kirkove C, Budach V, Malngon P, Valli MC (2013). Irradiation of the internal mammary and medial supraclavicular lymph nodes in stage I to III breast cancer: 10 years results of the EORTC Radiation Oncology and Breast Cancer Groups phase III trial 22922/10925 [Abstract]. Eur J Cancer.

[27] Konings AW, Smit Sibinga CT, Aarnoudse MW (1978). Initial events in radiation induced atheromatosis. Damage to intimal cells. Strahlen therapie.

